# A Study on Wireless Charging for Prolonging the Lifetime of Wireless Sensor Networks

**DOI:** 10.3390/s17071560

**Published:** 2017-07-04

**Authors:** Weijian Tu, Xianghua Xu, Tingcong Ye, Zongmao Cheng

**Affiliations:** 1 School of Computer Science and Technology, Hangzhou Dianzi University, Hangzhou 310018, China; 10924118@hdu.edu.cn (W.T.); tingcong.ye@hdu.edu.cn (T.Y.); 2 School of Science, Hangzhou Dianzi University, Hangzhou 310018, China; zmcheng@hdu.edu.cn

**Keywords:** mobile charger, network size, moving path, optimization problem, wireless energy transfer, wireless sensor network

## Abstract

Wireless charging is an important issue in wireless sensor networks, since it can provide an emerging and effective solution in the absence of other power supplies. The state-of-the-art methods employ a mobile car and a predefined moving path to charge the sensor nodes in the network. Previous studies only consider a factor of the network (i.e., residual energy of sensor node) as a constraint to design the wireless charging strategy. However, other factors, such as the travelled distance of the mobile car, can also affect the effectiveness of wireless charging strategy. In this work, we study wireless charging strategy based on the analysis of a combination of two factors, including the residual energy of sensor nodes and the travelled distance of the charging car. Firstly, we theoretically analyze the limited size of the sensor network to match the capability of a charging car. Then, the networked factors are selected as the weights of traveling salesman problem (TSP) to design the moving path of the charging car. Thirdly, the charging time of each sensor node is computed based on the linear programming problem for the charging car. Finally, a charging period for the network is studied. The experimental results show that the proposed approach can significantly maximize the lifetime of the wireless sensor network.

## 1. Introduction

In recent years, many works have focused on employing wireless energy conversion technology to charge the nodes in wireless sensor networks [1]. The wireless charging models can be simply divided into point-to-point model [2,3,4,5] and point-to-multipoint model [6,7,8,9]. In the former case, a mobile charger (MC), or a charging car, can only charge a single sensor node. In the latter case, the MC can charge more than one sensor node simultaneously. In this paper, we focus on developing an optimal wireless charging strategy based on the point-to-point model to prolong the lifetime of sensor networks.

Many works have studied wireless charging based on the point-to-point model [2,3,4,5]. Peng et al. proposed a charging scheme based on the residual energy of sensor nodes [3]. However, such a method ignored the travelled distance of the MC. The extra travelled distance may waste energy of the MC. In [2,4,5], researchers employed the traveling salesman problem (TSP) to design the moving path of the MC. They selected the shortest distance between sensor nodes as the variable. However, it did not consider the factor of residual energy of nodes. Some works [2,3,10] utilized a charging strategy in which the charging car completely charges the sensor nodes one by one. Such a method did not arrange the charging time for each sensor node. It may result in that the sensor nodes with very low energy at the end of the moving path will turn off before the MC arrives.

The traveling salesman problem (TSP) is one of the most famous algorithms in the field of mathematics. Assuming that there is a traveler who wants to visit *N* cities, he has to choose a path to follow. The path constraint is that the traveler visits each city once, and returns to the starting point. The TSP algorithm aims to find out the shortest distance of all moving paths.

In this paper, we focus on developing a wireless charging strategy, which is called integrated path and charging time scheme (IPCTS), to prolong the lifetime of the sensor network. The IPCTS is studied based on the analysis of the travelled distance of MC and the residual energy of the sensor nodes. We theoretically analyze the limited size of the sensor network to match the charging capability of a MC. We consider the networked parameters as the inputs of a Hamilton circuit problem under a given networked size. For the charging time allocation problem, we transform the maximum network lifetime into charging time problem by using linear programming. The main contributions of this paper are as follows:

- We first theoretically analyze the limited size of the sensor network to match the charging capability of a MC.
- We consider two factors to design the moving path of the MC, including the residual energy of nodes and the distance between nodes.
- To maximize the lifetime of network, we utilize the linear programming to compute the charging time of each node.

The remainder of this paper is organized as follows: in Section 2, we review the related work on wireless sensor networks published in recent years. Section 3 introduces our model and some assumptions. In Section 4, the upper and lower limits of network size are analyzed. Section 5 designs the moving path of a MC by the TSP problem. Section 6 models the charging time and solves the optimization problem to obtain the charging time of each node. Section 7 introduces the charging period. Section 8 presents simulation results. Section 9 concludes this paper.

## 2. Related Work

Wireless power transfer technology can be divided into three categories: inductive coupling [11], microwave radiation [12] and magnetic resonance coupling [1]. In [13], Xie et al. were the first to employ wireless power conversion technology to prolong the lifetime of network by deploying a charging car into wireless sensor network. Since the wireless energy can be radiated in all directions and in a particular direction, the charging model of MC can be divided into point-to-point model and point-to-multipoint model.

Based on the point-to-point charging model, Peng et al. did some work on how to maximize the network lifetime in [3,14]. In [3], the authors first proved that the charging problem is a non-deterministic polynomial complete (NPC) problem. Then, they proposed two heuristic algorithms known as Greedy and GreedyPlus. The Greedy algorithm takes the remaining energy of sensor node ascending order as the charging sequence, and the required charging time to prolong network lifetime. The GreedyPlus takes a fixed time as the charging time for each sensor node by a binary search. However, these heuristic algorithms do not consider the travelled distance of the MC, which could increase the MC’s energy consumption and reduce the charging time for sensor nodes. In [14], the authors computed the charging time by a binary search. As for charging sequence, the authors analyzed three routing protocols: energy-minimum routing, energy-balanced routing and charging-aware routing. According to the routing information, the nodes with large energy consumption in the network are considered as the charging nodes; and the remaining energy ascending order is used as the charging sequence.

In [2,4,5], the authors minimized the number of MCs needed to ensure that the network could run persistently. In [2], the authors divided the whole network area into several sub-regions by a K-means clustering algorithm. Then, they dispatched a MC for each sub-region to charge the sensor nodes in the sub-region. In [5], the authors designed a Hamiltonian circuit between all nodes as the shortest charging path for the MC. Then, the charging path was divided into several sub-paths based on the limited energy of the MC. Finally each sub-path dispatched a MC. In [2], Liang et al. proposed a *q-root* TSP to dispatch multiple MCs, and minimize the number of deployed MCs. The authors first introduced a tree decomposition algorithm with a provable 5-approximation ratio to find *q* closed tours. Then, an approximation algorithm was proposed to minimize the number of MCs, according to the total distance of each tour. However, a drawback of this strategy is that the complexity grows exponentially with the number of to-be-charged nodes.

There was another study on maximizing network utility which is expressed as the amount of data collected in the network [15,16]. In [16], the authors first selected a subset of the sensor nodes as anchor. The sencar (called a multifunctional mobile entity) stops at the anchor position to charge the anchor and collect data. Besides, the authors proposed an anchor selection algorithm based on remaining energy to control the travelled distance of the sencar within a given threshold. In [15], the authors combined flow conservation, energy balance and battery capacity as the constraints to maximize the utility function and turn it into a non-convex optimization problem. Finally, the authors transformed the original problem into a convex optimization method by using auxiliary variables.

As for point-to-multipoint charging model, researchers [6,17,18] maximized charge efficiency. Xie et al. took the charge efficiency as an objective function and formulated a mixed nonlinear program [17,18]. In [18], authors first addressed an ideal case assuming zero traveling time for the charger. By adopting the discretization and logic point representation techniques, a provably near optimal solution is obtained. Based on this solution, the authors further obtained the moving path of the original problem by finding the shortest Hamiltonian cycle. With this moving path, a feasible solution is further derived. In [17], the authors solved the mixed nonlinear programming. The discretization and re-linearization techniques were used. The authors proved the error between the feasible solution and the optimal solution. In [6], Qin et al. used cellular network topology and the center of cell as the stop points for an MC. It can charge all sensor nodes in the cell. However, the authors do not illustrate the stop time of the MC at each stop point. With the increase of network size, the travelled distance will grow linearly because of increasing number of cells.

Besides the charging efficiency, [10] aimed to minimize the number the stop points of the MC. In order to decrease the stop points, the authors used the overlapped portion of the energy reception range of the node as the set of stop points for the MC and planned a Hamiltonian circuit in the set. At each stop point, the MC charges the nodes within its charging radius and then moves to the next stop point. In [19], the authors consider the limited total energy of the MC as a constraint to make more nodes be charged. Based on the point-to-multipoint charging model, the authors first optimized the moving path to reduce the travelled distance of the MC and proved that the path problem is a non-deterministic polynomial (NP) hard problem. Finally, the authors proposed a heuristic algorithm particle swarm optimization (PSO) algorithm to solve the moving path and maximize the number of charging nodes.

In [20], Naranjo et al. proposed a P-SEP algorithm to select optimal cluster heads based on heterogeneous sensor nodes. The cluster head transfers data to achieve energy load balancing. In [21], Ahmadi et al. aimed to use an efficient routing algorithm to reduce communication cost under the condition of maintaining *k*-coverage. In [22], Mostafaei et al. proposed an ICABC method based on the ICA algorithm. The authors aimed to use the minimum number of sensor nodes to guarantee barrier coverage. These approaches can prolong network lifetime by reducing the network energy consumption. Compared with these approaches, we focus on how to design the optimal charging algorithm.

In the previous work, [3] employed wireless charging to prolong the lifetime of the sensor network. However, as for the charging sequence, the authors used the remaining energy ascending order as the movement path of the MC, which only considers the energy factor of the nodes. The Greedy algorithm [3] completely charges sensor nodes one by one. The GreedyPlus algorithm charges the sensor nodes within a fixed time. In [14], authors combined the routing protocol to design the charging scheme. In [6,17], researchers used linear programming to compute the charging time based on a point-to-multipoint charging model.

## 3. Model and Hypothesis

In this work, we assume that there is a MC, a base station and *n* similar sensor nodes in the network. The base station can acquire the remaining energy and the energy consumption rate of the sensor nodes. It generates a charging strategy for the MC. The charging strategy includes the charging path of the MC and the charging time of each sensor node.

The MC moves with a constant velocity starting from the base station. Therefore, the energy consumption of the MC is only related to the moving distance. The maximum amount of power carried by MC is denoted as $E_{MC}$. When a charging period is finished, the MC will return to the base station to replenish its power. Assume that the output power of MC is $P_{out}$, and the wireless output range is fan-shaped, then only one node can be charged. The reception power of sensor node is denoted as $P_{in}$. The relationship between the output power of MC and the reception power of sensor node is [10]:

(1) $$P_{in}=P_{out}\times \mu (d)$$

In Equation (1), μ(d) is a function of the distance between the MC and sensor node within a range of [0, 1]. In this work, we assume that the MC moves closely to the sensor node, thus, μ(d)=1, $P_{in}=P_{out}$.

We randomly deployed n sensor nodes in a rectangular area of dimensions L×L in the network. The coordinates of the sensor nodes are denoted as $\left(x_{i},y_{i}\right)$. Each sensor node is equipped with a rechargeable battery with a maximum capacity of $E_{t}$. The energy consumption rate of node i is denoted as $C_{i}$ with a range of $\left[C_{\min},C_{\max}\right]$. The nodes are not equal to probability values. We assume that the energy consumption rate is subject to a Gaussian distribution. In the real scenario, the energy consumption rate of sensor nodes is mostly around the mean value. Only a small part is particularly high or low. Let the energy consumption rate of the sensor node be denoted as X, which is subjected to a Gaussian distribution with mean $\mu_{x}$ and variance $\sigma_{x}^{2}$. Table 1 describes the symbols used in this paper and their respective meanings.

## 4. Network Size Analysis 

We aim to maximize the network lifetime using only one MC in the sensor network. As the energy of this MC is limited, the network size cannot be too large. In this section, we analyze the size of the network with reasonable number of sensor nodes.

### 4.1. Maximum Network Size

We assume that there are *n* nodes which need to be charged in the network. The energy consumption rate $C_{i}$ of each node is in the range of $\left[C_{\min},C_{\max}\right]$, and the probability of $C_{i}$ is subject to a Gaussian distribution expressed as X, $X\sim Ν(\mu_{x},\sigma_{x}^{2})$. Similarly, the moving distance of the MC is expressed as Y, $Y\sim Ν(\mu_{Y},\sigma_{Y}^{2})$.

The actual energy consumption rate of each node is unknown before the network is running, but what is known is that the energy consumption rate is subject to a Gaussian distribution, so the probability of energy consumption rate in $\mu_{x}\pm \sigma_{x}$ is 68.5% and it can be up to 95.5% when the energy consumption rate value is $\mu_{x}\pm 2\sigma_{x}$.

Taking $\mu_{x}\pm \sigma_{x}$ as an example, the total energy consumption of the network is no more than the chargeable charging capacity of the MC. The chargeable charging capacity is defined as the remaining energy which the total energy carried by the MC minus the energy consumed by movement, that is, the total energy that the MC can deliver to the sensor nodes. Then the following inequality holds:

(2) $$\left(T-\frac{\left(n-1)\times (\mu_{Y}+\sigma_{Y}\right)}{v}\right)\times P_{out}\geq n\times (\mu_{X}+\sigma_{Y})\times T$$

In Equation (2), T is the charging period, v is moving speed of the MC, $P_{out}$ is the output power of the MC, $\mu_{Y}$ is the expected value of the moving distance of the MC and $\mu_{X}$ is the expected value of the sensor node consumption rate. Further, Equation (3) is obtained by transposition of Equation (2):

(3) $$n\leq \frac{P_{out}\times \left(v\times T+\left(\mu_{Y}+\sigma_{Y}\right)\right)}{T\times v\times \left(\mu_{X}+\sigma_{X}\right)+\left(\mu_{Y}+\sigma_{Y}\right)\times P_{out}}$$

The charging period consists of three parts: total moving time of the MC, $T_{travel}$, charging time of all sensor nodes, $T_{ch\arg e}$, and the time that the MC takes to go back to replenish its energy at the base station $T_{back}$. The charging period T can be denoted using Equation (4):

(4) $$T=T_{travel}+T_{charge}+T_{back}$$

where:

(5) $$\begin{array}{c} T_{travel}=\frac{\left(n-1\right)\times \left(\mu_{Y}+\sigma_{Y}\right)}{v}\\ \frac{n\times \left(\mu_{X}+\sigma_{X}\right)\times T}{P_{out}}\leq T_{ch\arg e}\leq \frac{E_{MC}-\left(n-1\right)\times \left(\mu_{Y}+\sigma_{Y}\right)\times C_{MC}}{P_{out}}\\ T_{back}=\frac{2L_{\max}}{v}+t_{schar} \end{array}$$

In Equation (5), we assume that the time for the MC to replenish its energy at the base station is a constant value, $t_{schar}$. $L_{max}$ is the maximum distance between the MC and the charging sensor node. According to the Equation (5), the charging period is given by the expression $T\leq \frac{\left(n-1\right)\times \left(\mu_{Y}+\sigma_{Y}\right)}{v}+\frac{E_{MC}-\left(n-1\right)\times \left(\mu_{Y}+\sigma_{Y}\right)\times C_{MC}}{P_{out}}+T_{back}$. Taking the charging period T to Equation (3), then the maximum network size $n_{up}$ is calculated as follows:

(6) $$n_{up}\leq \frac{-b+\sqrt{b^{2}-4ac}}{2a}$$

(7) $$\left\{ \begin{array}{c} a=\left(\mu_{Y}+\sigma_{Y}\right)\times \left(\mu_{X}+\sigma_{X}\right)\times \left(P_{out}-v\times C_{MC}\right)\\ b=\left(\mu_{X}+\sigma_{X}\right)\times v\times E_{MC}+\left(\mu_{X}+\sigma_{X}\right)\times v\times \left(\mu_{Y}+\sigma_{Y}\right)\times C_{MC}\\ -P_{out}\times \left(\mu_{Y}+\sigma_{Y}\right)\times \left(\mu_{X}+\sigma_{X}\right)+P_{out}\times v\times \left(\mu_{Y}+\sigma_{Y}\right)\times C_{MC}+v\times \left(\mu_{X}+\sigma_{X}\right)\times P_{out}\times T_{back}\\ c=P_{out}\times v\times \left(E_{MC}+\left(\mu_{Y}+\sigma_{Y}\right)\times C_{MC}+P_{out}\times T_{back}\right) \end{array}\right.$$

### 4.2. Minimum Network Size

**Figure 1:**
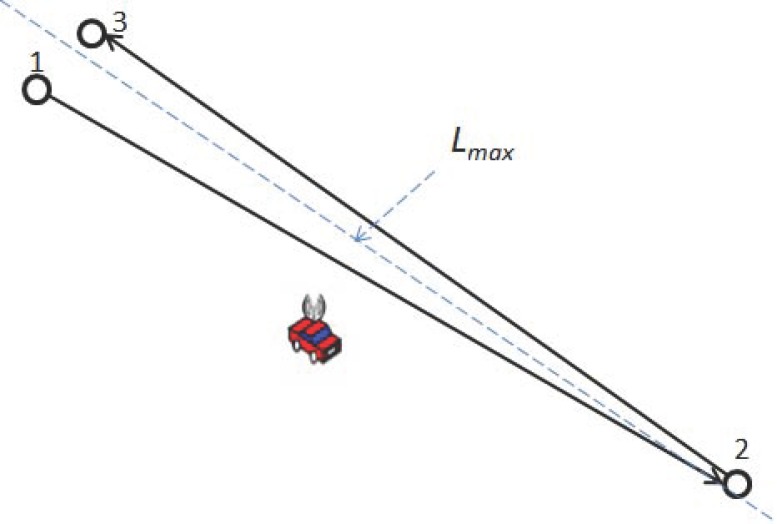
Situation where the MC must move the longest distance.

Assume that there are *n* nodes (hollow dots) in the network to be charged, as shown in Figure 1. The energy consumption rate of *n* nodes is maximized. The moving distance of the MC takes the max value as $L_{max}$ at each time.

According to that the total energy consumption of the network is less than the energy capacity of charging car, and we can get the following equation:

(8) $$n\leq \frac{P_{out}\times \left(v\times T\times L_{\max}\right)}{T\times v\times C_{\max}+L_{\max}\times P_{out}}$$

Further:

(9) $$n\leq \frac{P_{out}\times \left(v\times T+L_{\max}\right)}{T\times v\times C_{\max}+L_{\max}\times P_{out}}$$

While taking the charging time to Equation (9), the minimum network size $n_{low}$ can be obtained as follows:

(10) $$n_{low}\leq \frac{-b+\sqrt{b^{2}-4ac}}{2a}$$

(11) $$\left\{ \begin{array}{c} a=L_{\max}\times C_{\max}\times \left(P_{out}-v\times C_{MC}\right)\\ b=C_{\max}\times v\times E_{MC}+C_{\max}\times v\times L_{\max}\times C_{MC}\\ -P_{out}\times L_{\max}\times C_{\max}+P_{out}\times v\times L_{\max}\times C_{MC}+v\times C_{\max}\times P_{out}\times T_{back}\\ c=P_{out}\times v\times \left(E_{MC}+L_{\max}\times C_{MC}+P_{out}\times T_{back}\right) \end{array}\right.$$

### 4.3. Scale Analysis

In this section, we firstly analyze the worst charging situation, that is, when the moving distance of the MC reaches its maximum value at the same time as the energy consumption rate of each sensor. In this situation, we regard $n_{low}$ as the lower network size limit. When the number of sensor nodes is less than $n_{low}$, we can make the network run continuously with one MC.

Then, we analyze the normal charging situation. That is, the moving distance of the MC has a value $\mu_{y}\pm \sigma_{y}$, while the energy consumption rate of each sensor has the value $\mu_{x}\pm \sigma_{x}$. In this situation, we regard $n_{up}$ as the higher network size limit. According to the characteristics of the Gaussian distribution, the probability of the higher limit is as high as 68.5%. To improve the accuracy of the probability, we can take the energy consumption rate and the distance of MC value as μ±2σ. When the number of sensor nodes is greater than $n_{up}$, we cannot prolong the network lifetime significantly using one MC. When the number of sensor nodes is between $n_{low}$ and $n_{up}$, our charging scheme can prolong the network lifetime greatly.

## 5. Plan Charging Path

In this section, we plan the moving path of the MC, which is a charging sequence, denoted as $V=\left\{v_{1},v_{2},\cdots ,v_{n}\right\}$. The MC moves starting from the base station and comes back to replenish its energy. We set the base station as $v_{0}$, the charging sequence can thus be denoted as $V=\left\{v_{0},v_{1},v_{2},\cdots ,v_{n},v_{0}\right\}$. 

The problem when planning a moving path is equivalent to the charging sequence problem of sensor nodes. Therefore, the moving path plan problem can be transferred to the TSP problem. The traditional weight of a TSP considers the distance factor. In this work, we consider the distance and the remaining energy of sensor node as the weights to calculate the optimal path, and then transform it into a TSP problem.

We denote $V=\left\{v_{0},v_{1},v_{2},\cdots ,v_{n},v_{0}\right\}$ as the sensor nodes. The sensor network is represented in the form of a graph G=(V,E;v,e), where V is the set of charging nodes, E is the edge between sensor nodes, v is the weight of each sensor node, and e is the weight of the edge between sensor nodes.

Our goal is to find the best path in G. We make the weight of node be the remaining energy of the sensor node, and the weight of the edge to be the distance between the sensor nodes as follows:

(12) ω=α×v+(1−α)×e

In Equation (12), ω is the weight, v is the remaining energy of sensor node, and e is the distance between the sensor nodes. α is a constant between 0 and 1.

When α=0, the path we figured out is the shortest path. When α=1, the path figured out is the ascending sequence by remaining sensor node energy. Equation (12) can be represented as:

(13) $$\omega =\alpha \times \frac{\lambda_{i}}{c_{i}}+\left(1-\alpha \right)\times \frac{d_{i}}{v}$$

In Equation (13), $\lambda_{i}$ is the remaining energy of sensor node i, $c_{i}$ is the energy consumption rate of sensor node i, $d_{i}$ is the distance between sensor nodes, and v is the speed of the MC. Therefore, we transform the two different dimensions into one weight. After determining the weight, we can transform the path problem into a TSP problem.

## 6. Charging Time Plan

It is necessary to determine the charging time of each sensor node. Our goal is to maximize the network lifetime by ensuring that the remaining energy of all the sensor nodes at any time is greater than $E_{min}$. Thus, the charging time of a sensor node in a charging period should meet the following constraints:

1. The charging time of each node cannot be too long, otherwise, the remaining energy of the post-charged node will be less than $E_{min}$.
2. The charging time of each node cannot be too short; otherwise, the remaining energy of the previous-charged node may be less than $E_{min}$ at the end of the period.
3. The total energy demand for all sensor nodes should be less than the rechargeable battery of MC.

According to the three constraints above, we transform the charging time problem into an optimization problem.

### 6.1. Upper Limit of Charging Time

**Figure 2:**
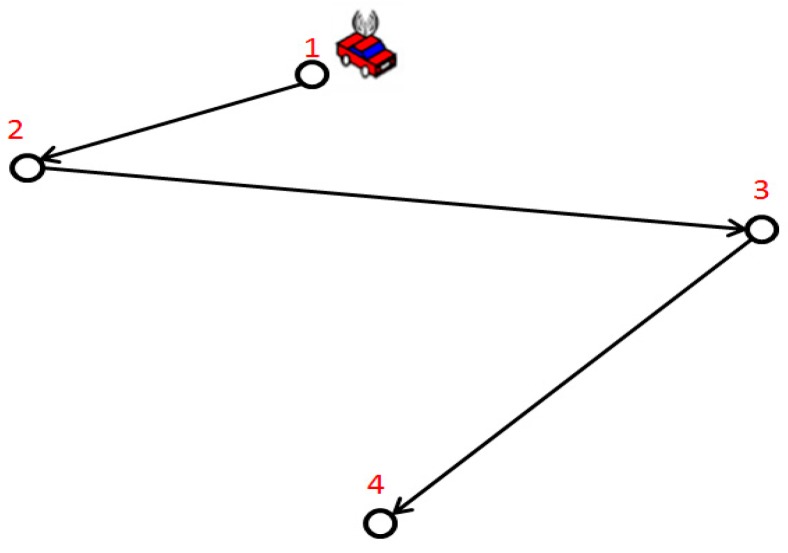
An example of a charging path.

As shown in Figure 2, the car is a MC. The hollow circles are sensor nodes which need to be charged. The charging sequence is denoted as $V=\left\{v_{1},v_{2},v_{3},v_{4}\right\}$. We set the charging time of sensor node as $t_{i}$. The constraint is that $t_{i}$ cannot be too large, otherwise, the remaining energy of the post-charged node will be less than $E_{min}$.

Take node 2 as an example, in Equation (14), when the MC arrives at node 2, the remaining energy of node 2 must more than the given threshold, as follows:

(14) $$\lambda_{2}-\left(\frac{d_{1,2}}{v}+t_{1}\right)\times C_{2}\geq E_{\min}$$

In Equation (14), $t_{1}$ is the charging time of sensor node 1, $\lambda_{2}$ is the remaining energy of sensor node 2, $C_{2}$ is the energy consumption rate of sensor node 2, and $d_{1,2}$ is the distance between sensor node 1 and 2. Further, the constraint condition can be denoted as Equation (15):

(15) $$\lambda_{i}-\left(\frac{\sum_{j=0}^{i-1}d_{j,j+1}}{v}+\sum_{j=0}^{i-1}t_{j}\right)\times C_{i}\geq E_{\min},i=1,...,|V|$$

where, |V| is the set of charging nodes.

### 6.2. Lower Limit of Charging Time

The charging time of each node cannot be too short. Otherwise, the energy of previous-charged sensor node will be less than $E_{min}$ at the end of a period. As shown in Figure 2, taking node 1 as an example, the remaining energy of node 1 must be more than the given threshold at the end of charging period, as follows:

(16) $$\lambda_{1}+t_{1}\times P_{out}-\left(t_{2}+t_{3}+t_{4}+\frac{d_{1,2}+d_{2,3}+d_{3,4}}{v}\right)\times C_{1}\geq E_{\min}$$

Further, the constraint condition can be denoted as Equation (17):

(17) $$\lambda_{i}+t_{i}\times P_{out}-\left(\sum_{j=i+1}^{\left|V\right|}t_{j}+\frac{\sum_{i}^{|V|-1}d_{i,i+1}}{v}\right)\times C_{i}\geq E_{\min},i=1,...,|V|$$

### 6.3. Energy Constraint

We consider the total energy of the MC is limited. Therefore, the required energy to be charged of all sensor nodes cannot be more than the remaining energy of the MC, and is limited as shown by Equation (18):

(18) $$P_{in}\times \sum_{i=1}^{\left|V\right|}t_{i}\leq E_{MC}-n\times L_{\max}\times C_{MC}$$

In Equation (18), $E_{MC}$ is the total energy of MC, $C_{MC}$ is the energy consumed by the MC and $P_{in}$ is the received power of the sensor node.

### 6.4. Problem Formulation

In order to maximize the lifetime of the sensor network, the MC should deliver the energy to the sensor nodes under the constraint that the remaining energy of each sensor node should always be more than $E_{min}$. Therefore, maximizing the charging time of each sensor node becomes the objective function. We transform the charging time problem into an optimization problem as follows:

(19) $$\max\overset{\left|V\right|}{\underset{i=1}{\sum }}t_{i}$$

(20) $$\left\{ \begin{array}{c} \lambda_{i}-\left(\frac{\sum_{j=0}^{i-1}d_{j,j+1}}{v}+\sum_{j=0}^{i-1}t_{j}\right)\times C_{i}\geq E_{\min}\\ \lambda_{i}+t_{i}\times P_{out}-\left(\sum_{j=i+1}^{\left|V\right|}t_{j}+\frac{\sum_{i}^{|V|-1}d_{i,i+1}}{v}\right)\times C_{i}\geq E_{\min}\\ P_{in}\times \sum_{i=1}^{\left|V\right|}t_{i}\leq E_{MC}-n\times L_{\max}\times C_{MC}\\ i=1,...,|V|\\ d_{0,1}=0,t_{0}=0 \end{array}\right.$$

In the optimization process, we need to analyze the feasibility region of the charging time. Considering the best charging path on which the moving distance of the MC is zero, which means $L_{\max}=0$. Take it to the Equation (18), the upper bound of feasibility region is $t_{upper}\leq \frac{E_{MC}}{n\times P_{in}}$. Due to the fact the charging time cannot be a negative number, the lower bound of the feasibility region should be greater than zero, $t_{lower}\geq 0$. Therefore, the charging time feasibility region is $\left[0,\frac{E_{MC}}{n\times P_{in}}\right]$.

### 6.5. Complexity Analysis

Our IPCTS approach includes charging path design and charging time computation. For the charging path design part, we solve the TSP problem by using a greedy algorithm to get a Hamiltonian circuit. To solve TSP, we calculate the distance between the each node and the charging car. It is therefore the complexity in the charging path design part is $O(n^{2})$. For the charging time computation part, the complexity is also $O(n^{2})$. Therefore, the overall complexity of IPCTS is $O(n^{2})$.

## 7. Charging Period Determination

In [15], the charging period of the MC is a constant value T, but in fact, the charging period is a changeable variable. If the charging period T is too short, the MC may not have enough time to charge all sensor nodes. On the contrary, if the charging period is too long, the MC may stay at the base station for a long time. Therefore, we do not make the charging time a constant in this work.

The charging period consists of three parts: total moving time of the MC, total charging time of all sensor nodes and the rest time of the MC at base station, which are denoted as $T_{travel}$, $T_{ch\arg e}$ and $T_{back}$, respectively. We denote the $k^{th}$ period as $T^{k}$, and get the following equation:

(21) $$T^{k}=T_{travel}^{k}+T_{charge}^{k}+T_{back}^{k}$$

Let a set of charging nodes are V={Φ}, the sensor nodes in V are sorted by their remaining energy from low to high, $s_{1},s_{2},\ldots ,s_{n}$. Assume that the node set is N, the number of nodes which need not to be charged is N−V, and they should meet the constraint as follows:

(22) $$\sum_{i\in |N|-|V|}\left(\lambda_{i}-C_{i}\times T^{k}\right)>E_{\min},i=1,...,|N|-|V|$$

This means that even if the sensor nodes in the set of N−V are not charged in the $T^{k}$ period, the remaining energy of the nodes within the set is more than the given threshold $E_{min}$ at any time.

1. Let $V=V\cup \left\{s_{1},s_{2}\right\}$, calculate $T_{travel}^{k}$, $T_{charge}^{k}$ and $T_{back}^{k}$ according $s_{1},s_{2}$, then calculate $T^{k}$.
2. Determine whether *V* satisfies the Equation (22)
3. If *V* satisfies the Formula (22), let $V=V\cup \{S_{i+1}\}$ and turn to 1, if not, turn to 4.
4. Return V and $T^{k}$.

## 8. Simulations

In this section, we demonstrate the effectiveness of our charging strategy through simulations. We explore the influence of parameters on the network lifetime by different experimental parameters. In [3], in order to prolong the network lifetime, Peng et al. proposed a heuristic algorithm called Greedy. The Greedy algorithm firstly gives a constant *k*, and the remaining energy of the lowest *k* nodes can be charged at each period. The charging sequence is sorted by *k* nodes in ascending order with remaining energy. In addition, the MC charges *k* nodes for each period. However, the optimal value of constant *k* is not easy to prove. The authors showed by experiments that the optimal value is 5. In this work, we also set the value of *k* be 5.

### 8.1. Parameter Settings

We deploy the sensor nodes randomly in a network. The number of sensor nodes increases from 40 to 70. The area is set from 50 m × 50 m to 200 m × 200 m. Other experimental parameters are set as shown in Table 2.

### 8.2. Result

The experimental platform is Eclipse Java EE IDE for Web Developers. All simulation results are averaged 100 times. Firstly, we randomly deploy 50 nodes in a 50 m × 50 m area. The relevant information of each node is shown in Table 3. According to the theoretical analysis and calculation, the upper and lower limits of the network size are 5 and 71, respectively.

**Figure 3:**
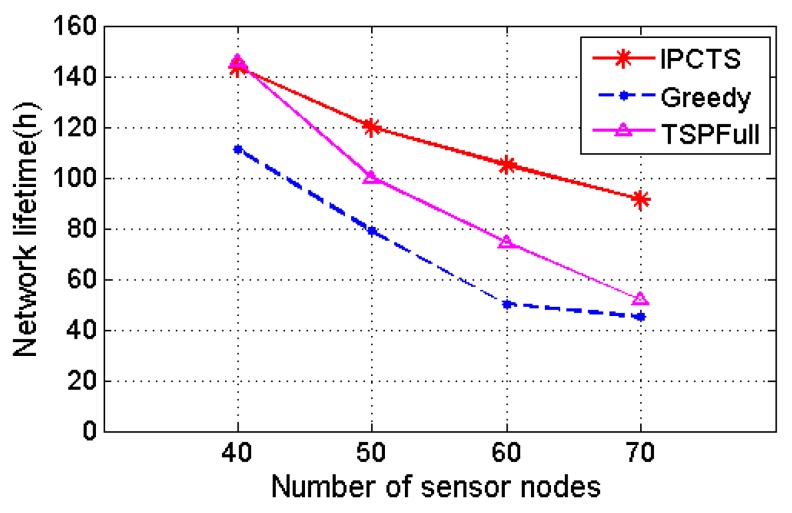
The effect of different charging times on the network lifetime.

Figure 3 shows the effect of different charging schemes on the network lifetime, when the number of nodes increases from 40 to 70 in the 100 m × 100 m area. TSPFull is a charging scheme which takes the Hamilton circuit as the charging path and uses the node energy to calculate the charging time. It can be seen that as the number of nodes increases, the network lifetime is gradually shortened. In contrast to Greedy Algorithm, IPCTS has a similar network lifetime as the Greedy one. However, with the network size gradually expanding, the IPCTS’s network lifetime is longer than those of the other two charging schemes. This is because of the fact that with the increase of the number of sensor nodes, the Greedy algorithm and TSPFull only charge five nodes, and most of the other nodes do not get an opportunity to charge. Therefore, their lifetime falls faster.

**Figure 4:**
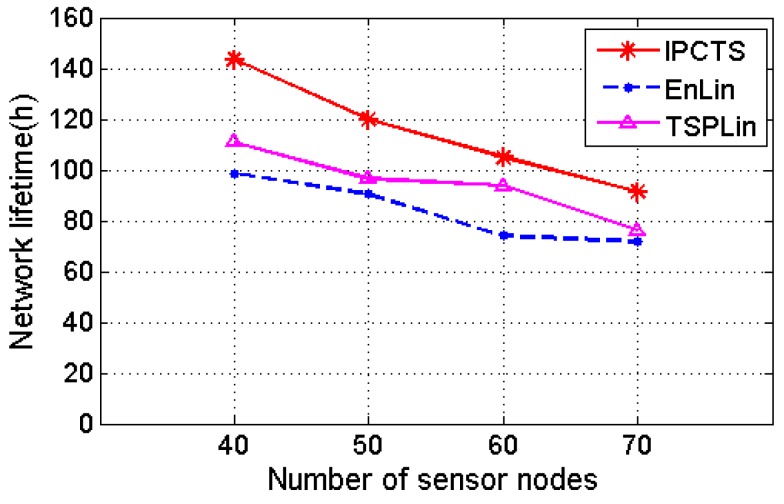
The effect of different charging paths on the network lifetime.

Figure 4 also shows the effect of different charging schemes, when the number of nodes increases from 40 to 70 in the 100 m × 100 m area. EnLin is a charging scheme which takes the ascending of remaining energy as the charging path of the MC and calculates the charging time of each node using linear programming. TSPLin considers the Hamiltonian circuit as the charging path, and uses linear programming to calculate the charging time of each node. Although the three charging schemes use linear programming to compute the charging time for each sensor node dynamically, they are different in moving path designs. Figure 4 shows that with the increasing number of nodes, the network lifetime of the three charging schemes decreases. The IPCTS considers the remaining energy of node and the length of charging path of MC, and its network lifetime is longer than those of the other two charging schemes.

**Figure 5:**
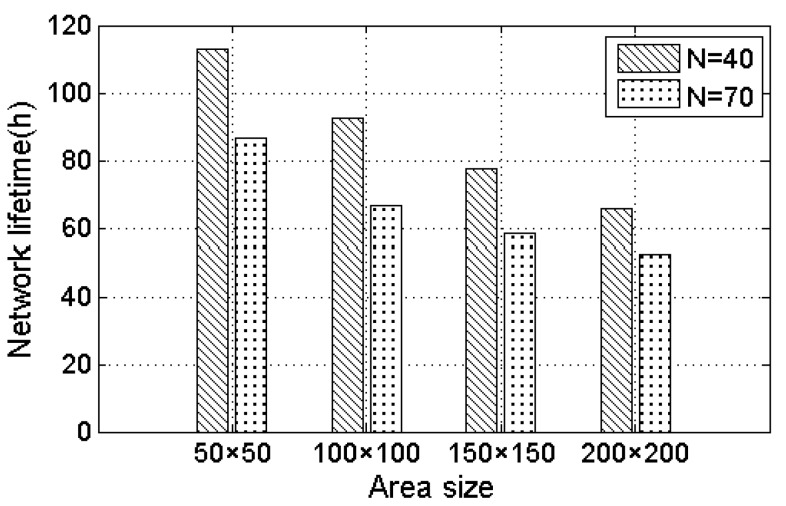
The effect of different area sizes on the network lifetime.

**Figure 6:**
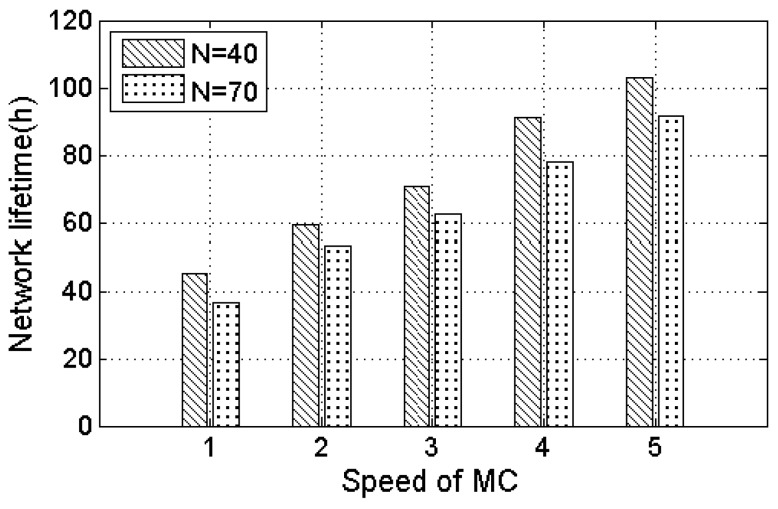
The effect of different moving speeds of the MC on the network lifetime.

Figure 5 shows the effect of different area size on the network lifetime. The number of nodes is selected to be 40 and 70 in this simulation. As can be seen from Figure 5, with the increase of the area, the network lifetime gradually reduces. This is because that with the increasing area size, the distance between the nodes increases, resulting in an increase in the moving path length of the MC. In addition, the MC consumes more energy on the moving path. Figure 6 shows the effect of different moving speeds of the MC to prolong the network lifetime. The area is 100 m × 100 m. In Figure 6, with the moving speed increasing, the network lifetime increases gradually. This is because of the fact that in the case of a constant area size, the reduction of moving time can bring an increase for charging time, and save MC energy consumption. Therefore, the network can get more energy replenished, and the fewer the number of nodes, the longer the lifetime.

## 9. Conclusions

In this paper, we explore how to charge sensor nodes through a MC in a wireless rechargeable sensor network to prolong the network lifetime. Since our MC has limited energy, we theoretically analyze the size of the network that a single MC can serve. We propose a charging scheme called IPCTS under a given network size to deal with the problem of charging sequence and the charging time. For the charge sequence problem, we plan the charging path as a classic TSP problem. For the charging time problem, we guarantee that the remaining energy of sensor node is greater than a given threshold at any time and transformed it into an optimization problem. The simulation experiments show that our charging scheme can significantly prolong the network lifetime.

## Figures and Tables

**Table 1:** Summary of notations.

Symbol	Meaning
$E_{t}$	total battery capacity of sensor node
$\lambda_{i}$	remaining energy of sensor node *i*
$P_{out}$	output power rate of MC
$P_{in}$	received power rate of sensor node
V	set of charging nodes
$C_{i}$	energy consumption rate of sensor node *i*
$s_{i}$	sensor node *i*
$C_{MC}$	moving energy consumption of MC
v	velocity of MC
$E_{MC}$	total energy of MC
n	number of sensor node
$n_{up}$	maximum network size
$n_{low}$	minimum network size
T	charging period
$T_{charge}$	charging time of MC
$T_{travel}$	moving time of MC
$T_{back}$	rest time of MC at base station
$t_{schar}$	the time to replenish electricity of MC
$E_{min}$	remaining energy threshold
$d_{ij}$	distance between sensor node *i* and node *j*
$t_{i}$	charging time of sensor node *i*

**Table 2:** Experimental parameter setting.

Parameters	Value
Total energy of MC: $E_{MC}$	10,000 J
Power out rate of MC: $P_{out}$	5 J/s
Moving consumption of MC: $C_{MC}$	30 J/m
Speed of MC: v	5 m/s
Initial energy of sensor node: $E_{t}$	1000 J
Mean and variance of Gaussian distribution	X~N (3, 1)
energy consumption rate range of node (mj)	(0, 6)

**Table 3:** Node coordinates and energy consumption rate.

Node ID	Coordinate (m)	Energy Consumption Rate (mj)	Node ID	Coordinate (m)	Energy Consumption Rate (mj)
0	(41.95, 3.56)	3.38	25	(7.39, 20.22)	3.67
1	(25.38, 0.78)	2.46	26	(25.38, 29.44)	2.85
2	(47.40, 34.26)	3.61	27	(21.45, 38.71)	2.49
3	(25.97, 47.81)	2.94	28	(4.27, 34.78)	2.88
4	(13.87, 1.48)	1.04	29	(14.06, 24.00)	1.65
5	(27.51, 29.14)	4.50	30	(19.38, 10.81)	3.04
6	(32.80, 42.98)	2.62	31	(21.51, 38.23)	3.57
7	(11.72, 6.57)	2.12	32	(49.64, 21.09)	3.23
8	(11.10, 10.76)	3.20	33	(27.82, 40.46)	4.31
9	(35.86, 44.93)	1.25	34	(31.20, 48.94)	3.01
10	(31.38, 43.16)	3.35	35	(25.50, 41.09)	1.77
11	(27.55, 34.19)	2.12	36	(11.61, 6.41)	3.90
12	(26.33, 14.06)	2.96	37	(8.02, 39.95)	3.55
13	(3.05, 32.65)	2.73	38	(47.20, 39.34)	2.13
14	(46.80, 10.19)	4.95	39	(38.88, 15.44)	3.16
15	(47.94, 13.53)	3.52	40	(11.92, 37.94)	3.38
16	(8.60, 47.63)	2.54	41	(38.83, 26.64)	3.18
17	(33.16, 31.55)	4.38	42	(21.79, 33.42)	2.17
18	(1.67, 4.09)	4.90	43	(26.61, 40.30)	2.65
19	(7.56, 26.88)	3.58	44	(41.52, 23.88)	3.76
20	(40.26, 12.48)	2.41	45	(11.21, 7.08)	2.57
21	(0.04, 14.33)	2.23	46	(2.31, 35.18)	3.29
22	(42.39, 47.80)	4.17	47	(31.17, 40.64)	3.86
23	(49.66, 12.87)	1.88	48	(32.04, 25.34)	2.55
24	(25.14, 20.98)	2.78	49	(6.55, 20.90)	1.66
